# The Two-Component System ArlRS and Alterations in Metabolism Enable *Staphylococcus aureus* to Resist Calprotectin-Induced Manganese Starvation

**DOI:** 10.1371/journal.ppat.1006040

**Published:** 2016-11-30

**Authors:** Jana N. Radin, Jessica L. Kelliher, Paola K. Párraga Solórzano, Thomas E. Kehl-Fie

**Affiliations:** 1 Department of Microbiology, University of Illinois Urbana-Champaign, Urbana, Illinois, United States of America; 2 Departmento de Ciencias de la Vida, Universidad de las Fuerzas Armadas ESPE, Sangolquí, Ecuador; National Institutes of Health, UNITED STATES

## Abstract

During infection the host imposes manganese and zinc starvation on invading pathogens. Despite this, *Staphylococcus aureus* and other successful pathogens remain capable of causing devastating disease. However, how these invaders adapt to host-imposed metal starvation and overcome nutritional immunity remains unknown. We report that ArlRS, a global staphylococcal virulence regulator, enhances the ability of *S*. *aureus* to grow in the presence of the manganese-and zinc-binding innate immune effector calprotectin. Utilization of calprotectin variants with altered metal binding properties revealed that strains lacking ArlRS are specifically more sensitive to manganese starvation. Loss of ArlRS did not alter the expression of manganese importers or prevent *S*. *aureus* from acquiring metals. It did, however, alter staphylococcal metabolism and impair the ability of *S*. *aureus* to grow on amino acids. Further studies suggested that relative to consuming glucose, the preferred carbon source of *S*. *aureus*, utilizing amino acids reduced the cellular demand for manganese. When forced to use glucose as the sole carbon source *S*. *aureus* became more sensitive to calprotectin compared to when amino acids are provided. Infection experiments utilizing wild type and calprotectin-deficient mice, which have defects in manganese sequestration, revealed that ArlRS is important for disease when manganese availability is restricted but not when this essential nutrient is freely available. In total, these results indicate that altering cellular metabolism contributes to the ability of pathogens to resist manganese starvation and that ArlRS enables *S*. *aureus* to overcome nutritional immunity by facilitating this adaptation.

## Author Summary

The ubiquitous pathogen *Staphylococcus aureus* is a serious threat to human health due to the continued spread of antibiotic resistance. This spread has made it challenging to treat staphylococcal infections and led to the call for new approaches to treat this devastating pathogen. One approach is to disrupt the ability of *S*. *aureus* to adapt to nutrient availability during infection. During infection, the host imposes manganese and zinc starvation on invading pathogens. However, the mechanisms utilized by *Staphylococcus aureus* to overcome this host defense are unknown. We report that ArlRS, a global staphylococcal virulence regulator, is important for resisting manganese starvation during infection. Loss of ArlRS does not prevent *S*. *aureus* from acquiring metals but instead renders the bacterium incapable of adapting to limited manganese availability. ArlRS mutants also have metabolic defects and a reduced ability to grow on amino acids. When using glucose as a carbon source *S*. *aureus* is more sensitive to manganese starvation and increases the expression of manganese transporters relative to when amino acids are provided suggesting a higher demand for manganese. These observations indicate that ArlRS contributes to resisting nutritional immunity by altering metabolism to reduce the staphylococcal demand for manganese.

## Introduction

*Staphylococcus aureus* is a ubiquitous pathogen that colonizes 30% of the population at any given time and can infect virtually every human tissue [1]. These facts and the continued spread of antibiotic resistance have led both the Centers for Disease Control and the World Health Organization to state that *S*. *aureus* poses a serious threat to human health [2, 3]. Both organizations have highlighted the need to identify new strategies for treating *S*. *aureus* and bacterial infections in general. Elucidating how pathogens overcome nutritional immunity, a critical component of the immune response in which the host restricts essential nutrients from the invading pathogen, has the potential to address this need.

Transition metals such as iron (Fe), manganese (Mn) and zinc (Zn) are essential for virtually all forms of life. Their importance is emphasized by the prediction that 30% of all enzymes interact with a metal cofactor [4, 5]. During infection, invading microorganisms must acquire all of their nutrients from the host. Vertebrates take advantage of this fact and combat invading pathogens by restricting the availability of essential metals [6, 7]. While the most well characterized aspect of nutritional immunity is the Fe-withholding response, it has recently become apparent that the host also restricts access to Mn and Zn during infection [7–12]. The prototypic example of Mn and Zn restriction is the staphylococcal abscess, which is rendered devoid of these two essential metals [8, 13]. This depletion starves *S*. *aureus* for these metals resulting in the inactivation of metal-dependent enzymes, such as the staphylococcal superoxide dismutases [8, 9]. A critical component of this withholding response is the Mn- and Zn-binding protein calprotectin (CP). CP comprises ~50% of the cytosolic protein in neutrophils and at sites of infection it can reach concentrations in excess of 1 mg/ml [14, 15]. Mice lacking CP have defects in metal sequestration and are more susceptible to a range of bacterial and fungal pathogens, including *S*. *aureus*, *Acinetobacter baumannii*, *Klebsiella pneumoniae*, and *Candida albicans* [8, 9, 16–19].

CP, a member of the S100 family of proteins, is a heterodimer comprised of S100A8 and S100A9 (also known as calgranulin A/calgranulin B and MRP8/MRP14), and has two transition metal-binding sites [9, 20]. The first site, known as the Mn/Zn site, is capable of binding either Mn or Zn with nanomolar and picomolar affinities (K_d_), respectively [9, 20–22]. The second site, known as the Zn site, binds Zn with picomolar affinity [9, 21–23]. CP exerts antimicrobial activity against a variety of bacterial and fungal pathogens *in vitro*, including *S*. *aureus*, by starving them for metals [8, 9, 16–19]. While the sequestration of both Mn and Zn contributes to the antimicrobial activity of CP, the Mn/Zn site is necessary for maximal antimicrobial activity by CP against a wide range of Gram-positive and Gram-negative pathogens including *S*. *aureus* [21].

The ability of *S*. *aureus* and other successful pathogens to cause disease indicates that they possess adaptations that allow them to minimize and overcome host-imposed Mn and Zn starvation. One mechanism employed by pathogens to cope with nutrient limitation is the expression of dedicated high-affinity acquisition systems. Mn and Zn import systems that contribute to pathogenesis are found in numerous pathogens including: *S*. *aureus*, *Brucella abortus*, *Campylobacter jejuni*, *Salmonella enterica*, *Yersinia pestis*, *Streptococcus pneumoniae*, *Streptococcus pyogenes*, *Streptococcus suis* and *A*. *baumannii* [6, 24–34]. *S*. *aureus* expresses two dedicated Mn import systems: MntH, an NRAMP family member, and MntABC, an ABC-type transporter [13, 31, 35]. In *S*. *aureus*, MntH is constitutively expressed, while MntABC is induced by Mn limitation [13, 31]. High-affinity Mn acquisition systems play a critical role in resisting Mn starvation during infection, and staphylococcal mutants lacking these systems are attenuated in several models of infection [13, 31, 36]. The virulence defect of a staphylococcal mutant lacking both Mn importers is reversed in CP-deficient mice, indicating that these systems specifically contribute to resisting host-imposed Mn starvation [13]. The ability of a mutant lacking dedicated Mn importers to cause comparable disease to wild type bacteria when Mn is not limited also highlights the critical importance of Mn sequestration to host defense.

While high-affinity metal acquisition systems contribute to infection, they do not prevent the host from imposing Mn starvation. This is evident by the increased bacterial burdens observed in CP-deficient mice and by inhibition of staphylococcal SOD activity during infection [8, 9]. *S*. *aureus* and other pathogens are able to successfully cause infection despite experiencing Mn and Zn starvation, thus they must possess additional adaptations that allow them to resist nutritional immunity. In this study, we identified the *S*. *aureus* two-component signal transduction system ArlRS as an important factor in resisting CP-imposed Mn starvation. Infection studies using wild type and CP-deficient mice revealed that ArlRS is necessary for establishing invasive *S*. *aureus* infection and resisting Mn starvation *in vivo*. Additionally, we discovered that *S*. *aureus* is more sensitive to Mn starvation when using glucose as a carbon source as compared to when amino acids are provided. Furthermore, ArlRS appears to play a critical role in facilitating the use of amino acids as a carbon source. These results indicate that altering core metabolic pathways is critical to overcoming host-imposed metal starvation.

## Results

### The *S*. *aureus* two-component system ArlRS promotes resistance to host-imposed manganese starvation

**Fig 1:**
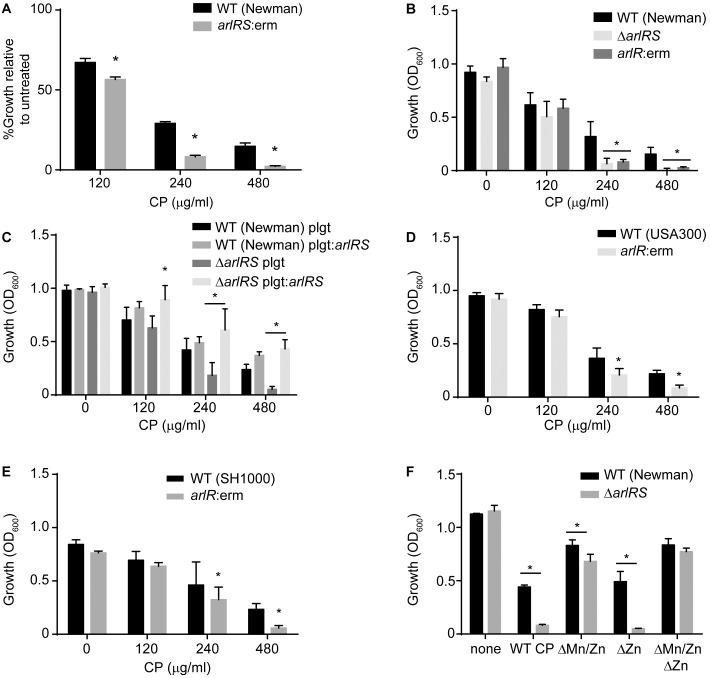
The *S*. *aureus* two-component system ArlRS plays a role in resisting calprotectin-imposed Mn starvation. Growth assays were performed in rich medium in the presence of increasing concentrations of CP for (A) WT (Newman) and the *arlRS*:erm derivative (Tn 917 transposon mutant), (B) WT (Newman), Δ*arlRS* and *arlR*:erm derivatives, (C) WT (Newman) and Δ*arlRS* containing either pOS1 plgt or pOS1 plgt:*arlRS*, (D) WT (USA300) and the *arlR*:erm derivative and (E) WT (SH1000) and the *arlR*:erm derivative. (F) Growth of WT *S*. *aureus* (Newman) and Δ*arlRS*, pre-cultured in NRPMI, in the presence of 480 μg/ml of WT CP, as well as the ΔMn/Zn, ΔZn and ΔMn/ZnΔZn site mutants. * = p≤0.05 by two-way ANOVA with Tukey’s posttests of selected means. n≥3. Error bars indicate SD. See also S1 Fig.

*S*. *aureus* experiences Mn and Zn starvation during infection, yet it is still able to successfully cause infection. This fact indicates that *S*. *aureus* possesses adaptations that allow it to overcome this host defense. To identify the factors that allow *S*. *aureus* to resist Mn and Zn starvation during infection, a transposon library was screened for mutants with enhanced sensitivity to CP. This screen identified a mutant that has an insertion in *arlRS* (*arlRS*:*erm*), which is more sensitive to CP than wild type *S*. *aureus* (Fig 1A). ArlRS is a two-component system and global virulence regulator that influences many staphylococcal processes, including autolysis, toxin expression, surface protein expression and biofilm formation [37–40]. As with most two-component systems, the signal sensed by ArlS is currently unknown. Subsequent assays using an ArlRS deletion mutant (Δ*arlRS*) and an *arlR* insertion mutant (*arlR*:erm) created in *S*. *aureus* Newman produced similar results to those obtained with the transposon mutant (Figs 1B and S1A). Similar to what was observed by Walker and colleagues, loss of ArlRS did not impact *hla* expression or hemolysis on blood agar plates [40] (S1B Fig). Expressing ArlRS from a plasmid reversed the increased sensitivity of Δ*arlRS* to CP (Fig 1C). Increased sensitivity to CP was also observed in *arlR*:erm derivatives of the community-acquired MRSA strain USA300 (JE2) as well as the methicillin-sensitive strain SH1000 (Fig 1D and 1E). In total, these results indicate that ArlRS promotes resistance to host-imposed metal starvation in both methicillin-sensitive and methicillin-resistant strains of *S*. *aureus*.

CP sequesters both Mn and Zn preventing the individual impact of withholding either metal from being evaluated with wild type protein. To circumvent this issue, the sensitivity of Δ*arlRS* to a series of engineered CP variants with altered metal-binding properties was assessed [9, 21]. Specifically, CP variants lacking the Mn/Zn site (ΔMn/Zn), the Zn site (ΔZn), or both sites (ΔMn/ZnΔZn) were utilized. When incubated with the ΔMn/ZnΔZn double site mutant, which cannot bind Mn or Zn, Δ*arlRS* grew as well as wild type (Figs 1F and S1C). This result confirms that the increased sensitivity of Δ*arlRS* to CP is due to an inability to cope with either Mn or Zn starvation. Similar to WT CP, Δ*arlRS* was more sensitive than wild type bacteria to the ΔZn site mutant, which can bind either Mn or Zn. However, the increased sensitivity of Δ*arlRS* was almost completely abrogated when grown in the presence of the ΔMn/Zn mutant, which can only bind Zn (Figs 1F and S1C). These results indicate that loss of ArlRS impairs the ability of *S*. *aureus* to cope with host-imposed Mn starvation.

### Autolysis does not contribute to the increased sensitivity to metal starvation

**Fig 2:**
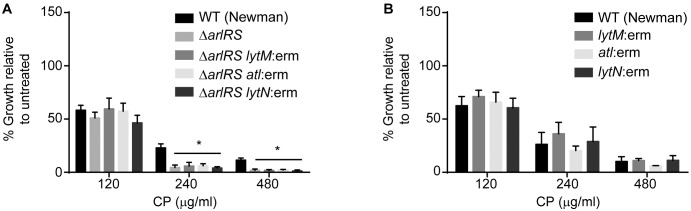
Autolysis does not contribute to increased sensitivity to metal starvation. The growth of (A) WT (Newman), Δ*arlRS*, Δ*arlRS/lytM*:erm, Δ*arlRS/atl*:erm and Δ*arlRS/lytN*:erm and (B) WT (Newman), *lytM*:erm, *atl*:erm, and *lytN*:erm were assessed in rich medium in the presence of increasing concentrations of CP. * = p≤0.05 by two-way ANOVA with Tukey’s posttests of selected means. n≥3. Error bars indicate SD.

ArlRS has been shown to repress expression of the staphylococcal autolysins LytM, LytN and Atl. As a result, loss of ArlRS can result in increased autolysis of methicillin-sensitive strains of *S*. *aureus* [38, 39, 41]. As such, cell lysis could potentially explain the enhanced sensitivity of Δ*arlRS* to CP. Previous studies revealed that the increased autolysis of strains lacking ArlRS can be reversed by individually deleting Atl or LytM in the Δ*arlRS* mutant background [41]. To determine if the diminished ability of Δ*arlRS* to resist Mn limitation is the result of increased autolysis, Δ*arlRS lytM*:erm, Δ*arlRS atl*:erm and Δ*arlRS lytN*:erm double mutants were assessed for CP sensitivity. Loss of LytM, LytN or Atl did not diminish the sensitivity of Δ*arlRS* to CP (Fig 2A). Control experiments revealed that loss of Atl, LytM, or LytN alone did not alter the sensitivity of *S*. *aureus* to CP (Fig 2B). These results indicate that increased sensitivity to CP of strains lacking ArlRS is not a result of increased autolysis. This idea is further supported by the increased sensitivity of the USA300 (JE2) *arlR*:erm mutant to CP (Fig 1D), as loss of ArlRS does not result in increased autolysis of methicillin-resistant isolates [41]. Cumulatively, these results indicate that increased autolysis does not contribute to the diminished ability of strains lacking ArlRS to resist Mn starvation.

### Loss of ArlRS does not reduce the ability of *S*. *aureus* to obtain Mn and Zn

**Fig 3:**
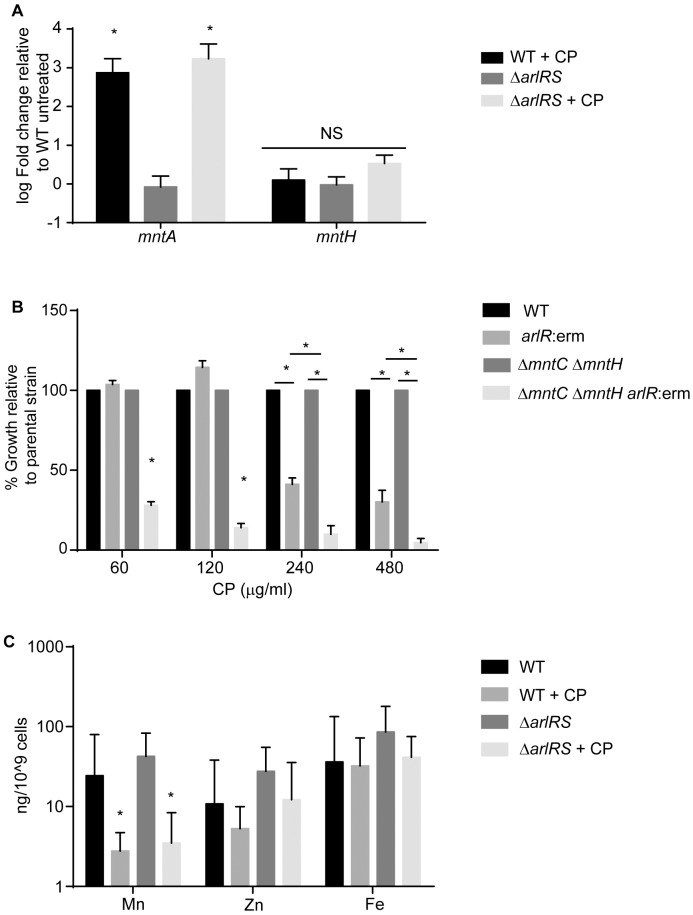
Loss of ArlRS does not alter Mn and Zn import. WT *S*. *aureus* (Newman) and Δ*arlRS* were grown in rich medium in the presence and absence of 240 μg/ml of CP. (A) Transcript levels of *mntA* and *mntH* were assessed by qRT-PCR. Expression was compared to wild type bacteria grown in the absence of CP. * = p≤0.05 by two-way ANOVA on log-transformed values with Sidak’s multiple comparisons test. n≥3. (B) WT *S*. *aureus* (Newman), *arlR*:erm, Δ*mntC* Δ*mntH* and Δ*mntC* Δ*mntH arlR*:erm were grown in the presence of increasing concentrations of CP. * = p≤0.05 by two-way ANOVA with Tukey’s posttests of selected means. n≥3. Strains are normalized to growth of parental strains, i.e., *arlR*:erm is normalized to WT and Δ*mntC* Δ*mntH arlR*:erm is normalized to Δ*mntC* Δ*mntH*. (C) Intracellular Mn, Zn and Fe levels were determined by ICP-OES. * = p≤0.05 by Student’s t-test. n≥3 Error bars indicate SD. See also S2 Fig.

Previous work demonstrated that loss of MntABC and MntH renders *S*. *aureus* twice as sensitive to CP as wild type bacteria [13]. Initially, to determine if the increased sensitivity of strains lacking ArlRS is due to decreased expression of Mn importers, the transcript levels of *mntA* and *mntH* were assessed by qRT-PCR. Following growth in metal-replete medium, wild type and Δ*arlRS* expressed comparable levels of both *mntA* and *mntH* (Fig 3A). Consistent with previous studies, CP treatment significantly increased *mntA* transcript levels in wild type bacteria (Fig 3A) [13]. CP also increased *mntA* expression in the strain lacking ArlRS, suggesting that the increased sensitivity of Δ*arlRS* is not due to reduced expression of Mn importers. We also assessed the impact that loss of ArlR had on a strain lacking the Mn importers (Δ*mntC*Δ*mntH arlR*:erm) to grow in the presence of CP. As before, the *arlR*:erm mutant was more sensitive to CP treatment than wild type bacteria (Figs 3B and S2). However, loss of ArlR in the Δ*mntC*Δ*mntH* mutant background further increased sensitivity of the transporter double mutant to CP, suggesting that ArlRS and the Mn transporters function independently to promote resistance to Mn starvation. To further evaluate if loss of ArlRS impacts the ability of *S*. *aureus* to acquire Mn or Zn, intracellular metal levels in wild type and Δ*arlRS* were directly assessed using inductively coupled plasma optical emission spectrometry (ICP-OES). This analysis revealed that loss of ArlRS does not impair Mn or Zn acquisition in the absence of CP (Fig 3C), as intracellular metal levels were the same in wild type bacteria and in Δ*arlRS*. In the presence of CP both WT and Δ*arlRS* had reduced levels of intracellular Mn (Fig 3C). No reduction in intracellular Zn or Fe were observed in the presence of CP. This observation is consistent with prior studies, which indicated that Mn binding is necessary for maximal antimicrobial activity [21]. Combined, these results indicate that a defect in metal acquisition is not responsible for the increased sensitivity of Δ*arlRS* to host-imposed metal starvation, suggesting that loss of ArlRS prevents *S*. *aureus* from adapting to limited Mn availability.

### ArlRS is necessary for establishing invasive *S*. *aureus* disease and resisting Mn starvation during infection

**Fig 4:**
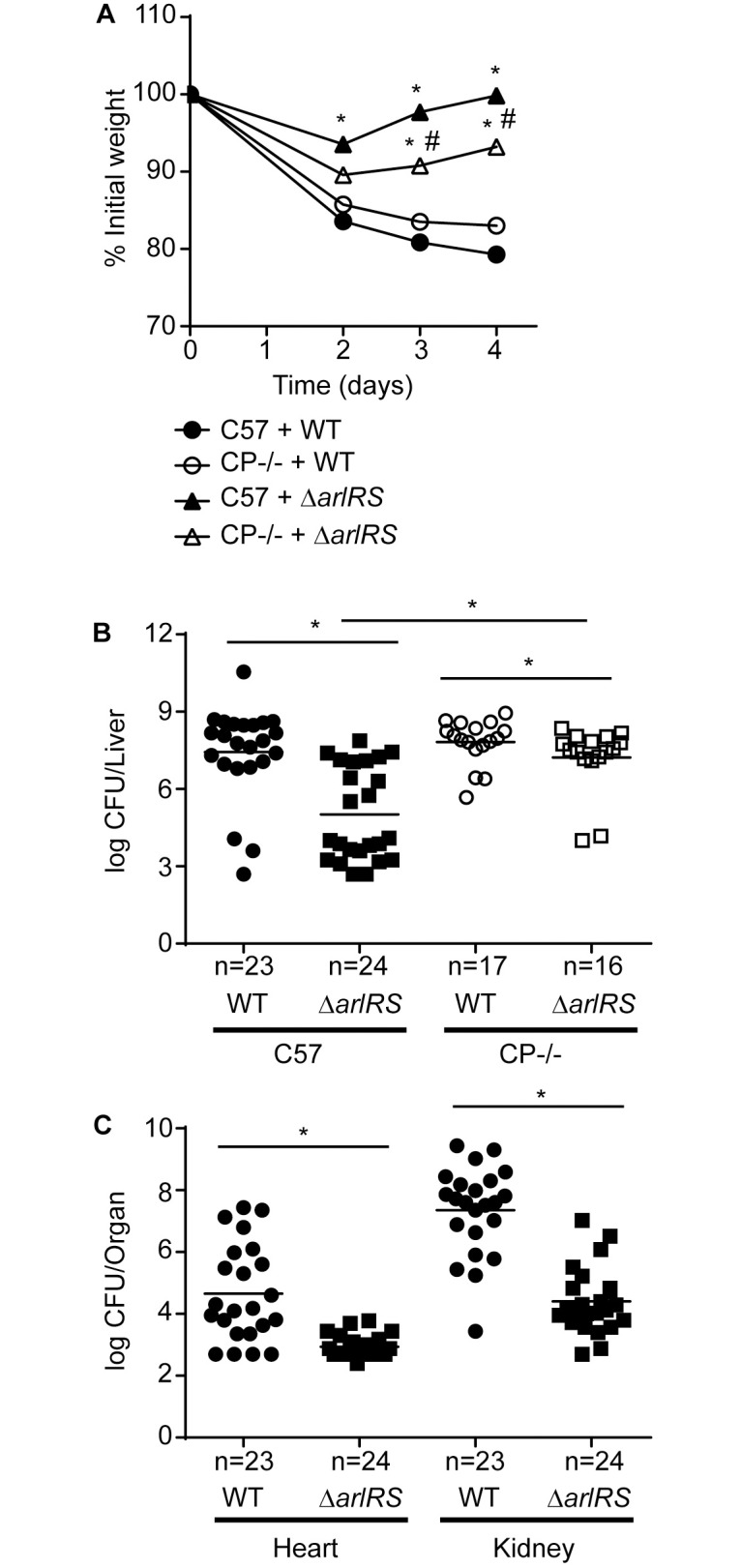
ArlRS is necessary for establishing invasive *S*. *aureus* infection and for resisting Mn sequestration during infection. Wild type C57BL/6 (C57) and CP-deficient C57BL/6 S100A9-/- (CP-/-) mice were infected with either *S*. *aureus* Newman (wild type) or Δ*arlRS* and (A) mean weight loss and (B-C) bacterial burdens in the (B) liver and (C) heart and kidneys were assessed after four days of infection. (A) See S3 Fig for pair wise comparisons that include error bars. * = p≤ 0.05 relative to C57BL/6 mice infected with wild type *S*. *aureus* and # = p≤ 0.05 relative to C57BL/6 mice infected with Δ*arlRS* by two-way ANOVA with Tukey’s posttest corrected for repeated measurements. (B-C) * = p≤0.05 as determined by Mann-Whitney test. The lines indicate the mean. The data are the results from three independent experiments. See also S3 Fig.

ArlRS contributes to hematogenous pyelonephritis and endocarditis in mouse and rabbit models of infection [39, 40, 42]. To determine whether ArlRS also contributes to systemic infection, wild type (C57BL/6) mice were retro-orbitally infected with wild type *S*. *aureus* Newman or Δ*arlRS*. During the course of the infection mice infected with Δ*arlRS* lost significantly less weight than mice infected with wild type *S*. *aureus* (Figs 4A and S3A). Consistent with the weight loss, the Δ*arlRS* mutant had significantly diminished bacterial burdens in the liver, heart, and kidneys when compared to wild type bacteria (Fig 4B and 4C) indicating that ArlRS plays an important role in establishing systemic disease. To evaluate the contribution of ArlRS to resisting Mn starvation during infection, CP-deficient (C57BL/6 S100A9-/-) mice, which do not remove Mn from liver abscesses [8, 13], were infected with wild type bacteria and Δ*arlRS*. Compared to C57BL/6 mice, the CP-deficient mice infected with Δ*arlRS* lost significantly more weight (Figs 4A, S3B and S3C). The CP-deficient mice infected with Δ*arlRS* also had increased bacterial burdens in the liver when compared to wild type C57BL/6 mice. Notably, despite the substantial virulence defect of the mutant in wild type mice, there was only a minimal difference between wild type *S*. *aureus* and Δ*arlRS* in the livers of CP-deficient mice (less than half a log difference vs. a 4 log difference). These results indicate that ArlRS contributes to systemic disease and that this two-component system is critical for resisting host-imposed Mn starvation during infection.

### Alteration in carbon source utilization promotes resistance to Mn starvation

**Fig 5:**
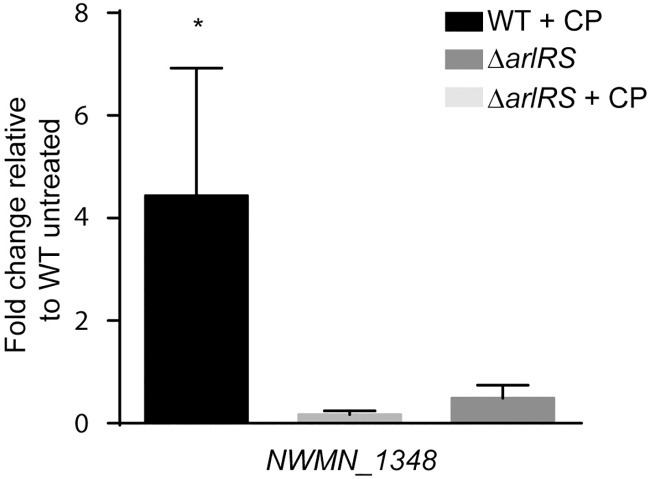
ArlRS enhances gene expression in response to calprotectin. WT *S*. *aureus* (Newman) and Δ*arlRS* were grown in rich medium in the presence and absence of 240 μg/ml of CP and transcript levels of the threonine/serine deaminase (NWMN_1348) was assessed by qRT-PCR. Expression was compared to wild type bacteria grown in the absence of CP. * = p≤0.05 by one-way ANOVA with Sidak’s multiple comparisons test. Error bars indicate SD. n≥3.

While the results so far demonstrate that ArlRS contributes to resisting host-imposed Mn starvation both in culture and during infection, the underlying mechanism is not apparent. ArlRS is a global regulator that is involved in many cellular processes including virulence factor gene regulation [37–39]. It is unlikely that the regulation of toxins and other virulence factors whose targets are absent in media would have an effect on resisting metal limitation in culture. In addition to controlling virulence factor expression, ArlRS negatively regulates the expression of genes encoding for several phosphotransferase systems (PTS) and positively regulates the expression of enzymes potentially involved in amino acid utilization [39]. This includes a locus that encodes for a putative alanine dehydrogenase, threonine/serine deaminase, and amino acid importer. This locus is induced upon exposure to CP and this induction is dependent on ArlRS (Fig 5). Glucose and other sugars are the preferred carbon source utilized by *S*. *aureus* and many other bacteria to generate energy [43, 44]; however, energy can also be generated using amino acids. While the metal dependency of glycolytic enzymes in *S*. *aureus* is unknown, Mn is a critical cofactor involved in sugar utilization by both *Bradyrhizobium* and *S*. *pneumoniae* [45, 46]. In contrast to sugars, amino acids can bypass the potentially Mn-dependent steps of glycolysis by being directly converted to pyruvate or TCA cycle intermediates [44, 47, 48]. Cumulatively, these observations lead to the hypothesis that Mn and Zn starvation may impair glycolysis. Furthermore, they suggest that the carbon source utilized could impact staphylococcal CP sensitivity and that ArlRS contributes to resisting Mn limitation by shifting *S*. *aureus* away from using sugars as an energy source to amino acids. If this hypothesis is correct, *S*. *aureus* would be expected to be more resistant to CP-induced Mn sequestration when using amino acids as opposed to glucose as a carbon source. Furthermore, loss of ArlRS would be expected to reduce the ability of *S*. *aureus* to grow when amino acids are provided as the sole carbon source and alter staphylococcal metabolism when both nutrient types are present.

**Fig 6:**
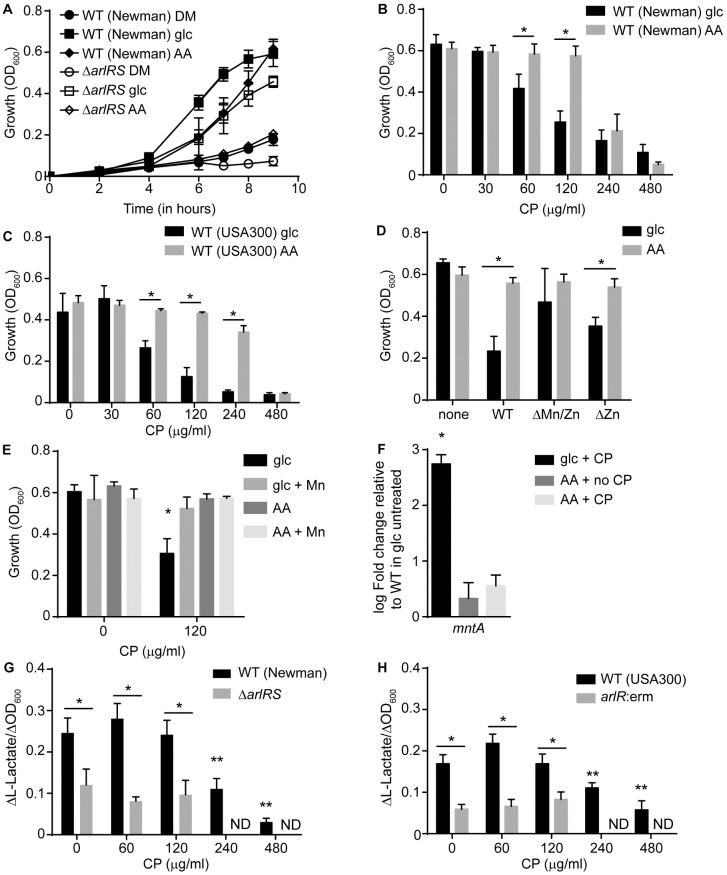
Alteration in carbon source utilization promotes resistance to Mn starvation. (A) Growth of *S*. *aureus* WT (Newman) and Δ*arlRS* in defined medium lacking a carbon source (DM) and medium supplemented with glucose (glc) or casamino acids (AA). (B-D) Growth assays were performed in defined medium containing either glucose (glc) or casamino acids (AA) as the sole carbon source in the presence of increasing concentrations of CP for (B) Newman, (C) USA300 (JE2), and (D) Newman with 120 μg/ml of WT CP, the ΔMn/Zn mutant or the ΔZn mutant. * = p≤0.05 by two-way ANOVA with Bonferroni’s posttest corrected for repeated measurements. n≥3. (E) WT (Newman) was grown in defined medium containing either glucose (glc) or casamino acids (AA) as a carbon source in the presence and absence of 120 μg/ml of CP and in the presence and absence of 500 μM MnCl_2_. (F) WT (Newman) was grown in defined medium containing either glucose (glc) or casamino acids (AA) as a carbon source in the presence and absence of 120 μg/ml of CP and transcript levels of *mntA* were assessed by qRT-PCR. Expression was compared to wild type bacteria grown in glucose-containing medium in the absence of CP. * = p≤0.05 by one-way ANOVA on log-transformed values with Dunnett’s multiple comparisons test. n≥3. L-Lactate production was measured for bacteria pre-cultured in NRPMI and assayed in defined medium containing both glucose and casamino acids in the presence of increasing concentrations of CP for (G) WT (Newman) and Δ*arlRS* and (H) WT (USA300 (JE2)) and *arlR*:erm. ND = not determined due to insufficient growth. * = p≤0.05 by one-way ANOVA on log-transformed values with Dunnett’s multiple comparisons test. n≥3. Error bars indicate SD for all panels except panels G and H, in which error bars indicate SEM. See also S4 Fig.

To test this hypothesis, a defined medium compatible with CP growth assays, which allows the carbon source to be altered, was developed (Fig 6A). This medium was then used to assess the sensitivity of *S*. *aureus* to CP when glucose or casamino acids were provided as the sole energy source. These assays revealed that *S*. *aureus* Newman is more sensitive to CP when glucose was provided as the sole carbon source compared to bacteria using casamino acids (Figs 6B and S4A). Similar results were also observed when a defined medium containing purified amino acids as an energy source was used (S4B Fig). USA300 (JE2) was also more sensitive to CP when only glucose was available as a carbon source. (Figs 6C and S4C). To determine whether the increased sensitivity of *S*. *aureus* to CP when glucose is used as a carbon source is dependent on Mn or Zn sequestration, the experiment was also performed with the ΔMn/Zn and ΔZn site mutants. While decreased growth in glucose-containing medium was observed when bacteria were growing in the presence of the ΔZn mutant (binds both Mn and Zn), growth in the presence of the ΔMn/Zn mutant (binds only Zn) was comparable to that of growth in medium containing amino acids, meaning that no growth defect was observed (Fig 6D). Additionally, the addition of excess Mn to glucose-containing medium reversed the phenotype (Fig 6E). Combined, these results suggest that Mn sequestration is responsible for the reduced ability of *S*. *aureus* to grow in glucose-containing medium in the presence of CP. If using glucose as a carbon source requires more Mn than amino acids, *mntA* expression would be expected to be higher in medium containing only glucose as an energy source relative to medium containing amino acids when Mn availability is restricted. Consistent with our hypothesis, *mntA* levels increased in the presence of intermediate concentrations of CP, but not in Mn-replete medium, when bacteria were grown in the presence of glucose but not in amino acids (Fig 6F). Cumulatively, these results indicate that utilizing glucose as carbon source increases the cellular demand for Mn when compared to when amino acids are used. Vitko et al. have previously shown that lactate is produced when bacteria are grown in glucose-containing medium but not when they are grown in amino acid-containing medium [49]. When Newman and USA300 (JE2) were grown in medium containing both glucose and amino acids in the presence of CP, lactate production decreased with increasing CP concentrations (Fig 6G and 6H). This observation is consistent with the idea that *S*. *aureus* shifts away from utilizing glucose as a carbon source when Mn is limiting and that utilizing amino acids as a carbon source minimizes the cellular demand for this metal.

Next, the ability of ArlRS mutants to utilize amino acids as an energy source was assessed. Analysis of the Δ*arlRS* derivative of Newman revealed that this strain was severely delayed in growth when utilizing amino acids as a sole carbon source (Figs 6A and S4D), suggesting a role for ArlRS in amino acid utilization. More modest but still significant reductions were also observed with *arlR*:erm derivatives of *S*. *aureus* USA300 (JE2) and SH1000 when only amino acids were provided as an energy source (S4E and S4F Fig). To evaluate if loss of ArlRS alters staphylococcal metabolism, the production of lactate was assessed in the Δ*arlRS* and *arlR*:erm derivatives of Newman and USA300 (JE2) following growth in the presence and absence of CP (S4G–S4J Fig). Both of the mutants had decreased lactate production relative to the parent strain regardless of CP treatment. Notably, differing from wild type, the strains lacking ArlRS did not reduce their production of lactate at inhibitory concentrations of CP (Fig 6G and 6H). In conjunction with the growth phenotypes, these results suggest that loss of ArlRS disrupts staphylococcal metabolism and results in reduced growth in the presence of amino acids as a carbon source. Combined, these results support the idea that switching from utilizing sugars to amino acids as an energy source reduces the staphylococcal demand for Mn enhancing the ability of *S*. *aureus* to resist host-imposed metal starvation. They also suggest that ArlRS critically contributes to this process.

## Discussion

Transition metals such as Fe, Mn and Zn are important for virtually all forms of life, as they are involved in numerous biological processes ranging from metabolism to regulation of virulence factor expression [4–6, 50]. To combat invaders, the host takes advantage of this essentiality by starving invaders for these metals. Recent work has revealed that in addition to restricting Fe availability, the essential transition metals Mn and Zn are also withheld by the host. Despite expressing high-affinity Mn acquisition systems, *S*. *aureus*, and presumably other successful pathogens, experience metal starvation during infection [8, 9]. However, the adaptations that allow pathogens to overcome host-imposed Mn and Zn starvation are unknown. The investigations in this study revealed that to successfully cope with host-imposed Mn starvation, *S*. *aureus* must alter core metabolic pathways. Previous studies have emphasized the importance of sugar consumption and fermentation to staphylococcal virulence [49, 51–54]. These obsevations include the finding that the catabolite control protein (CcpA), which promotes the consumption of sugars, and an expanded repertoire of glucose importers enhances the ability of *S*. *aureus* to cause disease [53, 54]. At the same time other studies sugest that uptake of amino acids facilitate the development of staphylococal disease [55]. In this study, we found that when bacteria encounter Mn starvation the presence of amino acids enhances the growth of the bacterium. These prior observations in conjunction with the current results emphasize the dynamic nature of sites of infection and further highlight the important contribution of metabolic plasticity to staphylococcal virulence and bacterial pathogenesis in general [49, 51, 52, 56, 57]. This work also revealed that the two-component system ArlRS enhances the ability of *S*. *aureus* to grow when amino acids are available as a sole carbon source and contributes to the ability of *S*. *aureus* to resist host-imposed Mn starvation during infection. This observation significantly expands the contribution of this two-component system to staphylococcal disease, which is canonically associated with regulation of toxin production and biofilm formation [37–40].

Recently, CP has been reported to bind Fe (II) with high affinity leading to the suggestion that the antimicrobial activity of the protein is derived from the ability to bind Fe, not Mn [58]. However, consistent with prior studies of *A*. *baumannii* [19], analysis of metal levels in *S*. *aureus* revealed that CP does not reduce intracellular Fe levels (Fig 3C). In contrast, in both S. *aureus* and *A*. *baumannii* CP reduced the accumulation of Mn [19]. These results suggest, at least for these two pathogens, that Fe sequestration is not a major contributor to the antimicrobial activity of CP. Additionally, the virulence defects in wild type mice of staphylococcal Δ*mntC*Δ*mntH* and Δ*arlRS* mutants, which are more sensitive to Mn starvation in culture, are reversed in CP-deficient mice [13]. These results, in conjunction with the inhibition of Mn-dependent enzymes during infection [9], further support the body of work indicating that Mn sequestration by CP contributes to host defense.

Canonically, glycolysis is thought to be a magnesium-dependent process. However, many bacteria contain a Mn-dependent isoform of phosphoglycerate mutase and other glycolytic enzymes such as enolase and pyruvate kinase that are either dependent on Mn or are highly activated by small amounts of Mn [24]. The increased sensitivity of *S*. *aureus* to Mn starvation when only glucose is available as a carbon source suggests that at least one essential step in glycolysis is dependent on Mn. This observation adds *S*. *aureus* to the growing list of organisms, including *S*. *pneumoniae* and *Bradyrhizobium japoincum*, which are dependent on Mn in order to consume glucose [45, 46]. The presence of Mn-utilizing glycolytic enzymes in a variety of microbes and the dependency of glycolysis in some pathogens on this metal suggests that host-imposed Mn starvation may also impede the ability of other pathogens to utilize sugars as an energy source.

**Fig 7:**
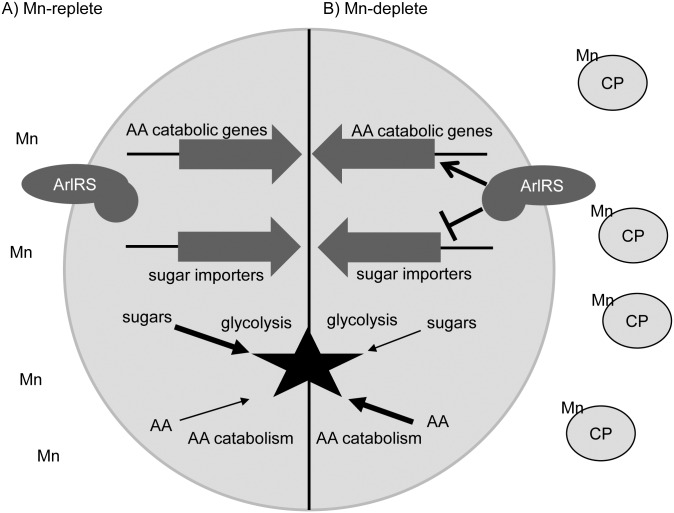
Working model of how calprotectin impacts staphylococcal metabolism and the contribution of ArlRS to resisting Mn limitation. In manganese (Mn)-replete environments (A) sugars serve as the primary energy source for *S*. *aureus*. However, in Mn-poor environments (B), such as in the presence of the Mn-binding protein calprotectin (CP), glycolysis is inhibited. ArlRS appears to enhance the ability of *S*. *aureus* to resist Mn limitation in part by increasing amino acid (AA) catabolic pathways and reducing the expression of alternative sugar importers.

The Fe-sparing response, the repression of Fe-rich enzymes when the availability of this metal is limited, enhances the ability of bacteria to grow in Fe-poor environments. This response allows bacteria to prioritize the usage of Fe by reducing the production of non-essential Fe-dependent proteins thereby preserving the limited quantity of available Fe for essential functions [59, 60]. The preferred carbon source of *S*. *aureus* and many other bacteria is glucose [43, 44]; however, energy can also be generated by utilizing amino acids. As such, glucose utilization is not strictly essential in *S*. *aureus*. In contrast to glycolysis, which can require Mn, the degradation of amino acids (e.g., alanine, serine and threonine) utilizes enzymes that do not employ this metal as a cofactor [44, 47, 48]. This observation suggests that relative to utilizing sugars, amino acid consumption should decrease the cellular demand for Mn, increasing the availability of this metal for essential Mn-dependent enzymes (Fig 7). Both the reduced expression of *mntABC* in Mn-limited medium when *S*. *aureus* is utilizing amino acids vs. glucose as a carbon source and the observation that growth on amino acids diminished staphylococcal sensitivity to Mn starvation support this idea. The latter observation suggests that consumption of amino acids instead of sugars is functionally a Mn-sparing response analogous to that of Fe. At higher concentrations of CP, *S*. *aureus* is equally sensitive to metal limitation regardless of whether the bacteria were grown in glucose- or amino acid-containing medium. Inhibition of Mn-dependent processes, which cannot be circumvented by switching carbon source utilization, could explain this observation. In response to Mn limitation, *S*. *pneumoniae* also downregulates glycolytic enzymes and increases the expression of amino acid utilization genes [46]. This observation and the current studies suggest that switching from utilizing sugars to amino acids is likely a conserved response to host-imposed Mn starvation.

While the utilization of amino acids as an energy source reduces the cellular demand for Mn, in most bacteria, including *S*. *aureus*, catabolite repression prevents them from utilizing non-preferred carbon sources, such as amino acids, when a preferred carbon source is present [61, 62]. ArlRS represses the expression of alternative sugar uptake systems and stimulates the expression of genes encoding for enzymes involved in amino acid utilization [39]. As such, ArlRS provides a mechanism by which *S*. *aureus* can override the normal carbon source preference of the cell. In response to CP, ArlRS positively regulates the expression of proteins predicted to be involved in the catabolism of alanine and serine. Differing from Liang et al, loss of ArlRS did not impact the expression of these proteins in the absence of CP. This difference can likely be explained by differences in growth conditions. These two amino acids can be converted directly to pyruvate, bypassing any metal-dependent enzymes in glycolysis [39, 44, 47, 48]. Notably, a global screen for staphylococcal factors that contribute to abscess formation identified alanine and serine importers as contributing to the ability of *S*. *aureus* to cause disease [55]. As with most two-component systems, the signal sensed by ArlS is currently unknown. The necessity of ArlRS to resist Mn starvation suggests that Mn availability alters the activity of this system. ArlRS may respond directly to Mn availability or indirectly by sensing a disruption of glycolysis or other Mn-dependent processes induced by Mn limitation. However, additional experimentation is necessary to evaluate this possibility.

ArlRS contributes to the ability of *S*. *aureus* to cause disease in several models of infection [42]. In addition to regulating metabolic processes, ArlRS increase the production of surface proteins and influences the expression of numerous virulence factors, biofilm formation, as well as autolysis and cell growth. It also directly and/or indirectly interacts with other regulators [37, 39]. Thus, a reduced ability to grow on amino acids may not be the only factor that contributes to the diminished ability of strains lacking ArlRS to resist CP. As ArlRS regulates the expression of other staphylococcal regulators, including Agr, LytSR, MgrA and Rot, the factors that are directly vs. indirectly regulated by this system are unknown [37–39]. While the direct targets are unknown, it does link Mn availability to virulence factor expression. Even though the benefit to *S*. *aureus* of co-regulating presumably Mn-independent virulence factors is not immediately apparent, this does appear to be a common theme amongst bacterial pathogens. In *Borrelia burgdoferi*, Mn influences BosR expression, which in turn regulates expression of the alternative sigma factor, RpoS. This alternative sigma factor facilitates the adaptation of *B*. *burgdoferi* to the mammalian host [63]. *S*. *pneumoniae* uses the Mn-responsive regulator PsaR to regulate the expression of adhesins [64–67].

Due to the continued emergence of antibiotic resistance, bacterial pathogens remain a serious threat to human health. The current study provides new insight into the mechanisms utilized by pathogens to overcome nutritional immunity. It suggests that alterations in carbon source utilization and reducing the cellular demand for Mn is important for resisting host-imposed Mn starvation. These results significantly broaden our understanding of how bacteria overcome nutritional immunity. The continued study of this bacterial response and the associated metabolic changes has the potential to identify new opportunities for therapeutic intervention.

## Materials and Methods

### Ethics statement

All experiments involving animals were reviewed and approved by the Institutional Animal Care and Use Committee of the University of Illinois Urbana-Champaign (IACUC license number 15059) and performed according to NIH guidelines, the Animal Welfare Act, and US Federal law.

### Bacterial strains

For routine overnight cultures, bacteria were grown in 5 ml of tryptic soy broth (TSB) or Chelex-treated RPMI plus 1% Casamino acids (NRPMI) supplemented with 1 mM MgCl_2_, 100 μM CaCl_2_ and 1 μM FeCl_2_ [13] in 15 ml conical tubes at 37°C on a roller drum. As needed, 10 μg/ml of chloramaphenicol was added for plasmid maintenance. *S*. *aureus* strain Newman and its derivatives were used for all of the experiments, unless otherwise indicated. For experiments using USA300 (JE2) and derivatives (USA300 (JE2) *arlR*:erm, USA300 (JE2) *lytM*:erm, USA300 (JE2) *lytN*:erm and USA300 (JE2) *atl*:erm), strains were obtained from the Nebraska library [68]. Newman Δ*arlRS* was generated by amplifying the 5’ and 3’ flanking regions (~1 Kb up- and downstream) of *arlRS* using the indicated primers (Table 1). 5’ and 3’ fragments were cloned into the pKOR1 knockout vector via site-specific recombination. The deletions were created using allelic replacement, as described previously [69]. All constructs were verified by sequencing and all mutant strains were confirmed to be hemolytic by growth on TSA blood agar plates. To generate constructs for complementation studies, the *arlRS* coding sequence was amplified with the indicated primers (Table 1) and cloned into the pOS1 vector under the control of the P_lgt_ promoter. The *lytM*, *atl* and *lytN* mutants in Newman and Newman Δ*arlRS* and *arlR* mutants in SH1000, Newman and Newman Δ*mntC*Δ*mntH* were constructed by transducing the *lytM*:erm, *atl*:erm, *lytN*:erm and *arlR*:erm alleles via Φ85 phage from USA300 (JE2) *lytM*:erm, USA300 (JE2) *atl*:erm, USA300 (JE2) *lytN*:erm and USA300 (JE2) *arlR*:erm.

**Table 1:** PCR primers used in this study.

Name	Sequence
ArlRS K/O 3’ Fwd	GGGGACAAGTTTGTACAAAAAAGCAGGCTCGTAATAGTACGTTGTAACATCGGTACAAGTGC
ArlRS K/O 3’ Rev	GGTGTACAAATGACGCAAATATTTTAATCATGACTGAGACGT CAATCAAAGTCATAGG
ArlRS K/O 5’ Fwd	CAGTCATGATTAAAATATTTGCGTCATTTGTACACCTCATATTACG
ArlRS K/O 5’ Rev	GGGGACTTTGTACAAGAAAGCTGGGTCGATAGAGAAAGACC TACATTGCTGCG
ArlRS 5’ Comp	AGTCCATATGACGCAAATTTTAATAGTAGAAGATGAAC
ArlRS 3' Comp	AGTCGGATCCTTAAAATATGATTTTAAACGTTGTTCCTTTG
16S rRNA-F	GCTGCAGCTAACGCATTAAGCACT
16S rRNA-R	TTAAACCACATGCTCCACCGCTTG
MntA RT Left	TCTAGATGAGCCGTTTGTCG
MntA RT Right	GCTTTTGATAGATCATGGTGGA
MntH RT Left	AATTCGATCATCGCAGTTCA
MntH RT Right	GCCACCTTGCATTGATGTTA
NWMN_1348 Fwd	GAACCATGCACAAGGTGTTG
NWMN_1348 Rev	ATAACCTTTGCCCCATAGCC
hla F	CTGAAGGCCAGGCTAAACCACTTT
hla R	GAACGAAAGGTACCATTGCTGGTCA

### CP growth assays

CP growth assays were performed, as described previously [9, 21]. Briefly, overnight cultures were back-diluted 1:50 into fresh TSB (5 ml in a 15 ml conical tube) and grown for 1 h at 37°C on a roller drum. The cultures were then diluted 1:100 into 96-well round-bottom plates containing 100 μl of growth medium (38% TSB and 62% calprotectin buffer (20 mM Tris pH 7.5, 100 mM NaCl, 3 mM CaCl_2_, 10 mM β-mercaptoethanol)) in presence of varying concentrations of CP. The growth medium was supplemented with 1 μM MnCl_2_ and 1 μM ZnSO_4_ except for assays with the Newman *arlRS*:erm transposon mutant. For all assays, the bacteria were incubated with orbital shaking (180 RPM) at 37°C and growth was measured by assessing optical density (OD_600_) every 2 hours. Prior to measuring optical density, the 96-well plates were vortexed. For CP growth assays using defined medium, a medium based on the one previously reported by Richardson et al. [51] was used. For these assays, the preculture was the same as a growth assay using TSB in the growth medium. The growth medium for assays using defined medium consisted of 38% medium and 62% CP buffer (20 mM Tris pH 7.5, 100 mM NaCl, 1 mM CaCl_2_, 10 mM β-mercaptoethanol). The defined medium (2.6X) consisted of 0.5 g/L NaCl, 1.0 g/L NH_4_Cl, 2.0 g/L KH_2_PO_4_, 7.0 g/L Na_2_HPO_4_, 0.228 g/L biotin, 0.228 mg/L nicotinic acid, 0.228 mg/L pyridoxine-HCl, 0.228 mg/L thiamine-HCl, 0.114 mg/L riboflavin, 0.684 mg/L calcium pantothenate, 0.104 g/L phenylalanine. 0.078 g/L lysine, 0.182 g/L methionine, 0.078 g/L histidine, 0.026 g/L tryptophan, 0.234 g/L leucine, 0.234 g/L aspartic acid, 0.182 g/L arginine, 0.078 g/L serine, 0.15 g/L alanine, 0.078 g/L threonine, 0.130 g/L glycine, 0.208 g/L valine and 0.026 g/L proline. The defined medium was then supplemented with 6 mM MgSO_4_, 1 μM FeCl_2_, 1 μM MnCl_2_ and 1 μM ZnSO_4_. Casamino acids (6.5%), glucose (1.3%) or glucose (1.3%) and 18 amino acids (1 mM alanine, serine, threonine, lysine, asparagine, glutamic acid, isoleucine, arginine, histidine, methionine, valine, proline, cystidine, glycine, phenylalanine, tyrosine, leucine and tryptophan) were provided as carbon sources as indicated. In the figures, “DM” refers to defined medium without a carbon source, “glc” refers to defined medium with glucose as a carbon source, “AA” refers to defined medium with casamino acids as a carbon source and “glc+18AA” refers to glucose and 18 amino acids as a carbon source. For complementation experiments, overnight cultures were back-diluted 1:50 into fresh TSB and grown for 2 h at 37°C [9, 21, 51]. When a metal starvation step was included the bacteria were grown overnight in NRPMI supplemented with 1 mM MgCl_2_, 100 μM CaCl_2_ and 1 μM FeCl_2_ and directly inoculated 1:100 in to the assay medium. Calprotectin was purified, as previously described [9, 21]. The initial ArlRS transposon mutant was identified during optimization experiments for screening a Tn917 mutant library. For these assays bacteria arrayed in 96-well plates were grown overnight in NRPMI supplemented with 1 mM MgCl_2_, 100 μM CaCl_2_ and 1 μM FeCl_2_. These cultures were then assayed for CP sensitivity, as described above.

### Expression analysis

To assess the expression of *mntA*, *mntH* and NWMN_1348, *S*. *aureus* was grown as for CP inhibition assays in complex medium in the presence and absence of 240 μg/ml of CP or in defined medium in the presence and absence of 120 μg/ml of CP. Bacteria were harvested during log phase growth (OD_600_ = ~0.1), samples were collected, an equal volume of ice-cold 1:1 acetone-ethanol was then added to the cultures, and they were frozen at -80°C until RNA extraction. RNA was extracted and cDNA was generated, as previously described [70–72]. Gene expression was assessed by quantitative reverse transcription-PCR (qRT-PCR) using the indicated primers (Table 1, [13]) and 16S was used as a normalizing control.

### ICP-OES analysis

To assess intracellular metal levels in wild type and Δ*arlRS*, *S*. *aureus* strains were grown as for CP inhibition assays using complex medium in the presence and absence of 240 μg/ml of CP. Bacteria were harvested during log phase growth (OD_600_ = ~0.1), washed twice with 0.1 mM EDTA, washed twice with water, and digested with nitric acid. Prior to nitric acid digestion an aliquot was used to determine the total number of bacteria in the sample. After digestion, ICP-OES was performed by the Microanalysis facility at the University of Illinois Urbana-Champaign.

### Animal infections

Mouse infections were performed, as described previously [8, 9], with the exception that mice were anesthetized with isoflurane. Briefly, 9-week old mice were infected retro-orbitally with approximately 1 x 10^7^ CFU in 100 μl of sterile phosphate-buffered saline. Following injection, the infection was allowed to proceed for 96 h before the mice were sacrificed. Livers, hearts and kidneys were removed, the organs were homogenized, and bacterial burden was determined by plating serial dilutions.

### L-Lactate assay

L-lactate production was assayed, as described previously [49]. Briefly, bacteria were grown as for CP inhibition assays described above using NRPMI overnight cultures and back-diluted 1:100 into defined medium in the presence and absence of 60, 120, 240 and 480 μg/ml of CP. Samples were harvested every hour during log phase, heat inactivated (70°C for 5 min), and supernatants were collected. Samples were stored at -20°C. L-Lactic acid concentrations were measured using a Roche Yellow Line kit (R-Biopharm).

## Supporting Information

S1 Fig ArlRS promotes resistance to host-imposed Mn starvation. (A) Growth of WT *S*. *aureus* (Newman), Δ*arlRS* and *arlR*:erm in the presence and absence of 240 μg/ml of CP. n≥3. (B) WT *S*. *aureus* (Newman) and Δ*arlRS* were grown in rich medium and transcript levels of *hla* were assessed by qRT-PCR. Expression was compared to wild type bacteria. n≥3. (C) Growth of WT *S*. *aureus* (Newman) and Δ*arlRS* pre-cultured in NRPMI in the presence of 240 μg/ml of CP and the ΔMn/Zn, ΔZn and ΔMn/ZnΔZn mutants. * = p≤0.05 by two-way ANOVA with Tukey’s posttests of selected means. n≥3. Error bars indicate SD.
(TIF)

S2 Fig Loss of ArlRS prevents *S*. *aureus* from adapting to limited Mn availability. WT *S*. *aureus* (Newman), *arlR*:erm, Δ*mntC* Δ*mntH* and Δ*mntC* Δ*mntH arlR*:erm were grown in the presence of increasing concentrations of CP. Growth was assessed by measuring optical density. n≥3.
(TIF)

S3 Fig ArlRS is necessary for establishing invasive *S*. *aureus* infection and for resisting Mn sequestration during infection. Graphs showing individual comparisons of the data presented in Fig 4A. Wild type C57BL/6 (C57) and CP-deficient C57BL/6 S100A9-/- (CP-/-) mice were infected with either *S*. *aureus* Newman (wild type) or Δ*arlRS* and weight loss was determined over time. (A) WT mice infected with wild type *S*. *aureus* vs. WT mice infected with Δ*arlRS*, (B) WT mice infected with Δ*arlRS* vs. CP-/- mice infected with Δ*arlRS*, and (C) CP-/- mice infected with wild type *S*. *aureus* vs. CP-/- mice infected with Δ*arlRS*. * = p≤ 0.05 by two-way ANOVA with Tukey’s posttest corrected for repeated measurements. Error bars indicate SD.
(TIF)

S4 Fig Growth in amino acid-containing medium promotes resistance to Mn sequestration. Growth assays were performed in defined medium containing either glucose (glc) or casamino acids (AA) as a carbon source in the presence and absence of 120 μg/ml of WT CP for (A) Newman and (C) USA300. (B) Growth of Newman in defined medium supplemented with glucose (glc) only or with glucose and 18 amino acids (glc+18AA) in the presence and absence of 60 μg/ml of WT CP. Growth in defined medium supplemented with casamino acids (AA) for (D) Newman and the Newman Δ*arlRS* derivative, (E) USA300 and the USA300 *arlR*:erm derivative and (F) SH1000 and the SH1000 *arlR*:erm derivative. * = p≤0.05 by two-way ANOVA with Bonferroni’s posttest corrected for repeated measurements. Growth in defined medium supplemented with glucose and amino acids in the presence and absence of CP for (G) Newman, (H) the Δ*arlRS* derivative, (I) USA300 and (J) the *arlR*:erm derivative. * = p≤0.05 by two-way ANOVA with Bonferroni’s posttest corrected for repeated measurements. n≥3. Error bars indicate SD.
(TIF)
