# Supplementary figures and images for: The Two-Component System ArlRS and Alterations in Metabolism Enable Staphylococcus aureus to Resist Calprotectin-Induced Manganese Starvation
Source: PLoS Pathog. 2016 Nov 30;12(11):e1006040. doi: 10.1371/journal.ppat.1006040 (PMC5130280; doi:10.1371/journal.ppat.1006040)

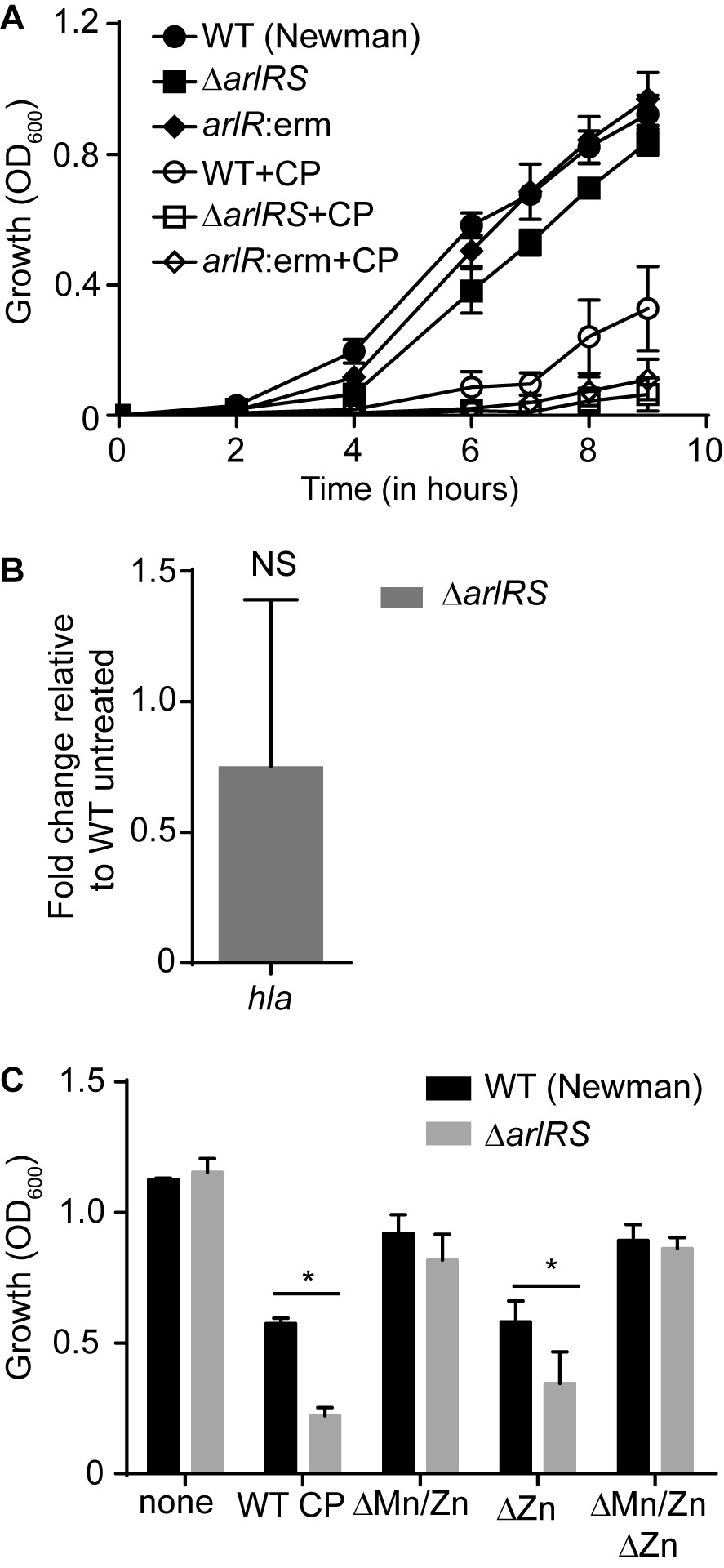

Supplement: S1 Fig — (A) Growth of WT S. aureus (Newman), ΔarlRS and arlR:erm in the presence and absence of 240 μg/ml of CP. n≥3. (B) WT S. aureus (Newman) and ΔarlRS were grown in rich medium and transcript levels of hla were assessed by qRT-PCR. Expression was compared to wild type bacteria. n≥3. (C) Growth of WT S. aureus (Newman) and ΔarlRS pre-cultured in NRPMI in the presence of 240 μg/ml of CP and the ΔMn/Zn, ΔZn and ΔMn/ZnΔZn mutants. * = p≤0.05 by two-way ANOVA with Tukey’s posttests of selected means. n≥3. Error bars indicate SD. (TIF) [file ppat.1006040.s001.tif]

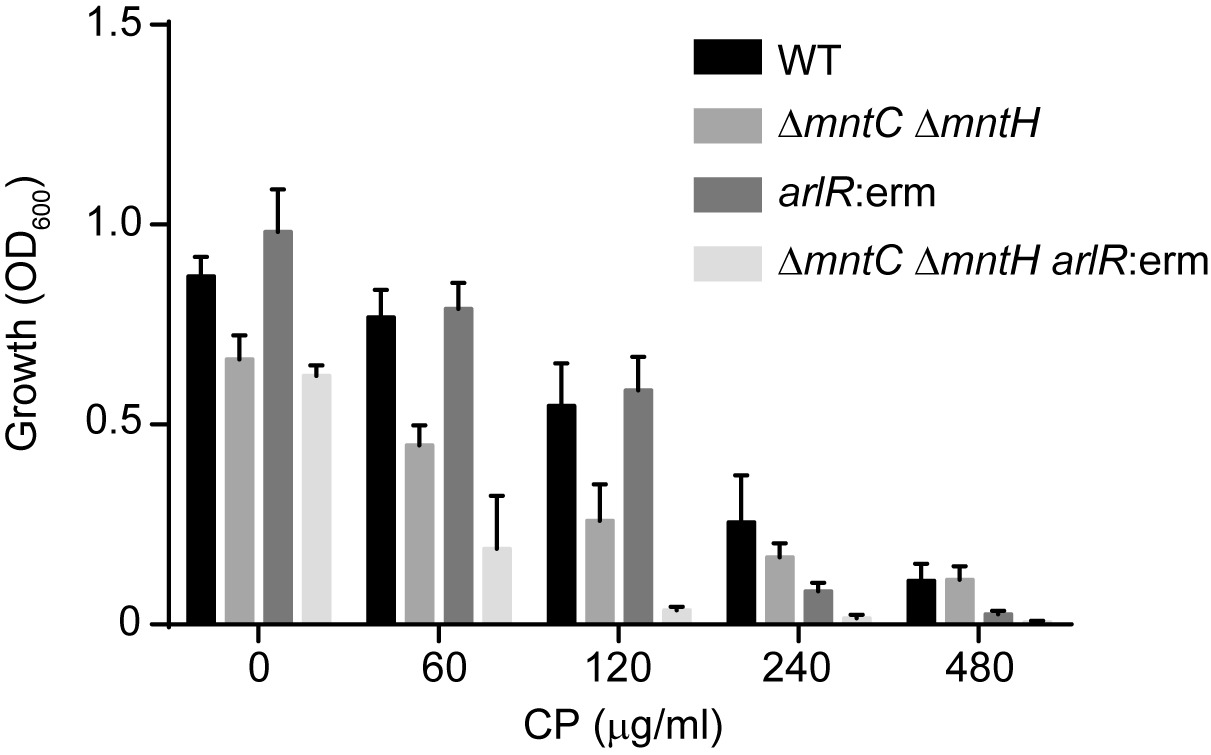

Supplement: S2 Fig — WT S. aureus (Newman), arlR:erm, ΔmntC ΔmntH and ΔmntC ΔmntH arlR:erm were grown in the presence of increasing concentrations of CP. Growth was assessed by measuring optical density. n≥3. (TIF) [file ppat.1006040.s002.tif]

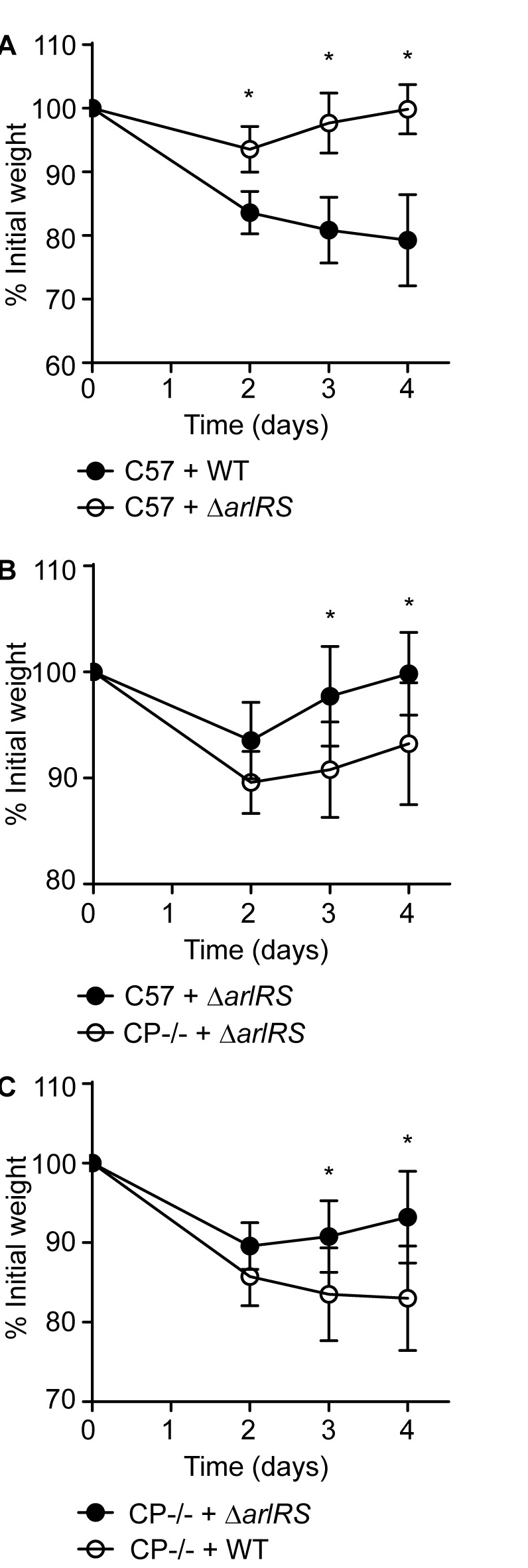

Supplement: S3 Fig — Graphs showing individual comparisons of the data presented in Fig 4A. Wild type C57BL/6 (C57) and CP-deficient C57BL/6 S100A9-/- (CP-/-) mice were infected with either S. aureus Newman (wild type) or ΔarlRS and weight loss was determined over time. (A) WT mice infected with wild type S. aureus vs. WT mice infected with ΔarlRS, (B) WT mice infected with ΔarlRS vs. CP-/- mice infected with ΔarlRS, and (C) CP-/- mice infected with wild type S. aureus vs. CP-/- mice infected with ΔarlRS. * = p≤ 0.05 by two-way ANOVA with Tukey’s posttest corrected for repeated measurements. Error bars indicate SD. (TIF) [file ppat.1006040.s003.tif]

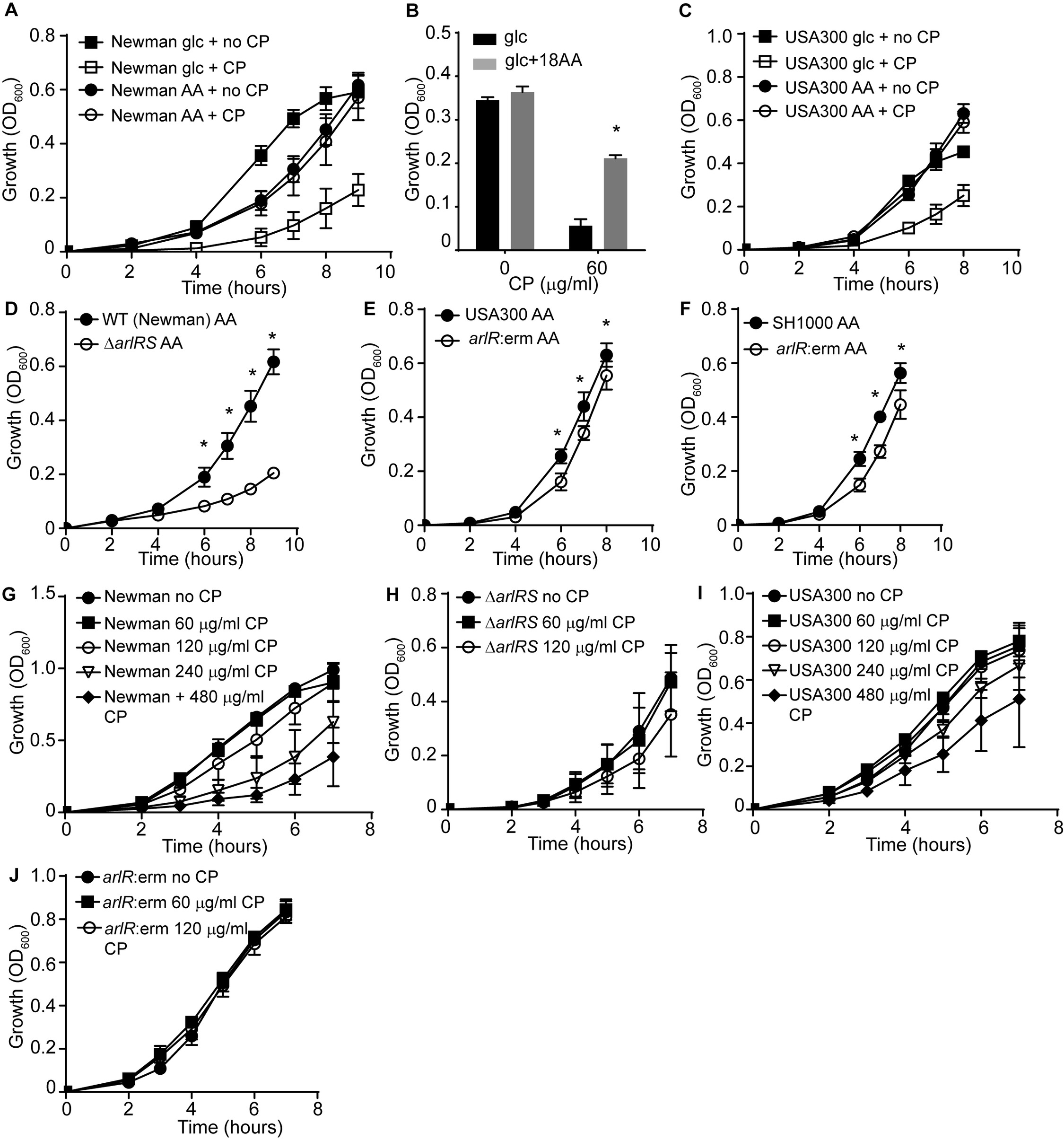

Supplement: S4 Fig — Growth assays were performed in defined medium containing either glucose (glc) or casamino acids (AA) as a carbon source in the presence and absence of 120 μg/ml of WT CP for (A) Newman and (C) USA300. (B) Growth of Newman in defined medium supplemented with glucose (glc) only or with glucose and 18 amino acids (glc+18AA) in the presence and absence of 60 μg/ml of WT CP. Growth in defined medium supplemented with casamino acids (AA) for (D) Newman and the Newman ΔarlRS derivative, (E) USA300 and the USA300 arlR:erm derivative and (F) SH1000 and the SH1000 arlR:erm derivative. * = p≤0.05 by two-way ANOVA with Bonferroni’s posttest corrected for repeated measurements. Growth in defined medium supplemented with glucose and amino acids in the presence and absence of CP for (G) Newman, (H) the ΔarlRS derivative, (I) USA300 and (J) the arlR:erm derivative. * = p≤0.05 by two-way ANOVA with Bonferroni’s posttest corrected for repeated measurements. n≥3. Error bars indicate SD. (TIF) [file ppat.1006040.s004.tif]
